# Acute Intestinal Obstruction: Notes on the Diagnosis and Treatment

**Published:** 1915-08

**Authors:** W. C. Gewin

**Affiliations:** Surgeon to the Birmingham Infirmary and Hillman Hospital. Birmingham, Ala.


### Journal-Record of Medicine

Successor to Atlanta Medical and Surgical Journal, Established 1855
and Southern Medical Record, Established 1870

**OWNED BY THE ATLANTA MEDICAL JOURNAL COMPANY**

PUBLISHED MONTHLY

*Official Organ Fulton County Medical Society, State Examing Board, Atlanta, Birmingham and Atlantic Railroad Surgeon's Association, Chattahoochee Valley Medical and Surgical Association, Etc.*

RICHARD R. DALY, M. D., Editor
BERNARD WOLFF, M. D., Supervising Editor
A. W. STIRLING, M. D., C. M., D. P. H.; J. S. HURT, B. Ph., M.D.
GEO. M. NILES, M. D., W. J. LOVE, M. D., (Ala.); Associate Editors
E. W. ALLEN, Business Manager

**COLLABORATORS**

W. F. WESTMORELAND, M. D., General Surgery
F. W. McRAE, M. D., Abdominal Surgery
ALLEN H. BUNCE, Medical Societies
E. B. BLOCK, M. D., Diseases of the Nervous System
MICHAEL HOKE, M. D., Orthopedic Surgery
CYRUS W. STRICKLER, M. D., Legal Medicine and Medical Legislation
E. C. DAVIS, A. B., M. D., Obstetrics
E. G. JONES, A. B., M. D., Gynecology
ALLEN H. BUNCE, M. D., Pathology and Bacteriology
R. T. DORSEY, JR., B. S., M. D., Medicine
L. M. GAINES, A. B., M. D., Internal Medicine
GEO. C. MIZELL, M. D., Diseases of the Stomach and Intestines
L. B. CLARKE, M. D., Pediatrics
EDGAR PAULLIN, M. D., Opsonic Medicine
THEODORE TOEPEL, M. D., Mechano Therapy
R. R. DALY, M. D., Diseases of the Eye, Ear, Nose and Throat
BERNARD WOLFF, M. D., Diseases of the Skin
E. G. BALLENGER, M. D., Diseases of the Genito-Urinary Organs

VOL. LXII. ATLANTA, GA., AUGUST, 1915. NO. 5

### ACUTE INTESTINAL OBSTRUCTION: NOTES ON THE DIAGNOSIS AND TREATMENT*

BY W. C. GEWIN, M. D.

Surgeon to the Birmingham Infirmary and Hillman Hospital.
Birmingham, Ala.

There is probably no darker chapter in surgery than that of acute intestinal obstructions, the mortality being estimated at

*Read before the Chattahoochee Valley Medical Asso'n, July 12-3, 1915, at Opelika, Ala.

anywhere from 60 per cent to 90 per cent. This is all the more deplorable when we take into consideration just what it represents. A very small part of this high rate of mortality is legitimate. The balance is due directly to delay. It is true that aside from strangulated hernia, rectal obstruction, and intussusception other forms of intestinal obstruction cannot be recognized with any degree of accuracy, nevertheless, it is possible to recognize that an obstruction is present in practically every case fairly early in its course. An exact distinction between the different forms of obstruction which demand immediate operation is in most instances unnecessary. However, it is essential to determine as clearly as possible the location and kind of lesion present in order to give the patient the advantage of an early operation, or to avoid an unnecessary one. Richardson says, "The diagnosis, therefore, between enteritis and peritonitis on one side and acute mechanical obstruction on the other is of extreme importance. Enteritis in the phlegmonous or ulcerative form occasionally resembles closely acute obstruction. In the former fever is present with gentral tenderness. The collapse is not extreme nor is the obstruction total. No tumor can be felt. Moreover, the history is of great significance, the symptoms appearing slowly and gradually increasing to their full development. Between acute general peritonitis and actte obstruction the diagnosis is sometimes impossible. Acute general peritonitis is always preceded by some exciting cause connected with the abdominal viscera from which inflammation extends into the general peritoneal cavity. If such causative lesions are present differentiation is usually easy; if they are absent the local and constitutional signs of general peritonitis may simulate so closely obstruction that a correct diagnosis is impossible. Indeed, even when a definite cause for peritonitis is known to exist it may be at times impossible to differentiate between these two conditions." In both acute mechanical obstruction and acute general peritonitis there are pain, distension, and vomiting, and as a rule complete constipation.

In the former condition the pain is in the region of the obstruction, and is violent, and paroxysmal in character; while in acute general peritonitis the pain usually radiates from the umbilicus or epigastrium, and is severe and constant. In acute obstruction before the development of peritonitis there is absence of muscular spasm, fever, and leucocytosis, but in peritonitis all three symptoms are usually present. When the lesion is above the iliocecal valve the evacuation of the contents of the lower bowel may occasion error in disagnosis. In the early stages of peritonitis there is usually expulsion of flatus and feces following enema which is not the case in acute obstruction.

In a large percent of cases it is necessary to perform an exploratory operation to determine the cause and location of the obstruction. Frequently the condition of the patient is so critical that one hesitates to perform an immediate operation, first attempting to stimulate the patient in order that he may be in better condition to stand the operation. Delay is seldom justifiable. It is at this stage that the bowel becomes rapidly gangrenous, and the condition of the patient progressively worse. Immediate operation with simultaneous administration of normal saline solution intravenously is in my mind the wisest course to pursue, unless the patient is already moribund.

With reference to the site of the incision, I use an opening through the right rectus muscle, unless I have a definite idea of the location of the lesion before opening the abdomen.

In that event I open immediately over the site of obstruction unless I have reasons to do otherwise. The site of the obstruction having been found and its cause determined, the question arises, "What is the best method of procedure in the particular type of tumor present?" A brief consideration of one or two of the most representative forms of obstruction will be given in order to elucidate a few practical ideas of the surgical treatment of these cases. In the surgical treatment of intussusception an idea that appeals to me as being very practical has been suggested by Beebe. After the intussusception has been reduced, he ligates and severs the meso-appendix, and returns the viscera to the abdominal cavity. "A blunt forceps is forced into the abdomen through a stab just within the iliac spine, the appendix caught at its tip and pulled through until the cecum is flush with the abdominal wall, and held there by a stitch through it and the skin. If there is much visceral distension the tip of the appendix is snipped off and a soft rubber catheter passed through its lumen into the small intestines, the finger guiding it through the illo-cecal valve. The appendicostomy affords a ready means of draining the bowel and introducing fluid for absorption. Should the distension prevent a ready return of the viscera into the abdominal cavity, it can be rendered easier by introducing the catheter first, and partially emptying the coils through it. Should drainage of the bowel not seem necessary at the time, it can still be utilized in the hours succeeding the operation should it become desirable. In any event it fixes the cecum firmly so that variety of intussusception cannot occur again and disposes of the appendix, which, at least sometimes, excites it. In a few days it can be snipped off flush with the skin, and soon disappears in the scar." If the intussusception proves to be irreducible or gangerous the outlook for the patient is much more grave. If the condition of the patient will admit of it, the tumor may be resected, and a Paul's tube tied into each end of the intestine, waiting until later to do an anastomosis. Frequently it is advisable to do nothing more than deliver the tumor from the abdominal cavity, pack gauze around it and open it, waiting until later to do an extra-peritoneal resection.

In obstruction resulting from carcinoma of the intestine unlike other forms of acute obstruction the first thing to determine is whether the growth can be completely removed, and whether there are metastatic foci. If the growth cannot be entirely removed, or if metastasis has already taken place, the operation should be made as simple as possible. Probably the best method of procedure is to bring out the distended bowel immediately above the growth, pack it off with gauze from the peritoneal cavity, open it, and tie in a Paul's tube. This opening may remain permanently as an artificial anus or if the condition of the patient warrants it a short-circuiting operation may be done, and the enterostomy opening closed, after the intestines have regained their normal tone. Short-circuiting is often done as a primary measure. This I believe in a great many instances is subject to criticism.

The inflamed and softened bowel is liable to give way at the line of suture. Also, the bacteria from the debris of an obstructed intestine are usually of a highly virulent character, and infection frequently follows anastomotic operations. In cases in which the growth is on the small intestine an artificial anus should not be maintained for any great length of time as it means starvation to the patient, and a severe irritation about the site of the exterostomy opening. In those cases in which removal of the growth is impossible there are several methods of operation available. If the patient is already emaciated and weak before the onset of the acute obstruction a radical primary operation should not be attempted. It is better to do an enterostomy above the growth and to resect and anastomose at a subsequent time, finally closing the artificial anus at a third operation. Where the condition of the patient will warrant it the growth may be resected, and a Paul's tube tied into each end of the divided gut. Later, anastomosis, and closure of the enterostomy will complete the operation.

As the guiding principles of the surgical treatment of all forms of acute intestinal obstruction are about the same, I have made no attempt to go into details, nor to consider all the forms of acute intestinal obstruction separately, however, I should like to mention a few salient points, with which you are no doubt familiar, but because of their importance deem them worthy of your attention, viz:

(1) Never delay operation, where mechanical obstruction of the bowel is present if there is the slightest chance of recovery for the patient.

(2) Lavage should be done as a routine measure before opening the abdomen.

(3) The intestines should be well protected at all times during the operation with warm saline sponges. The sponges should not be constantly changed, but should be kept warm by placing hotter sponges over them.

(4) Always empty the bowel above the constriction if there is much distension present.

(5) An artificial anus in the large bowel may remain for an indefinite length of time, but in the small intestine severe irritation soon results, and the patient soon starves.

(6) If a permanent artificial anus is to be maintained, it is best to make it as near the umbilicus as possible, as this is the most stationary point, as well as the most convenient place to retain a suitable dressing.

(7) In the small intestine an extensive resection and immediate anastomosis may be done with a great deal more impunity than in the large bowel.

(8) Each inch of the root of the mesentery nourishes about three feet of intestine, therefore care must be exercised not to destroy the blood supply beyond the proposed resection.

Birmingham Infirmary, Birmingham, Ala.

